# A Comparison Between GPT-3.5, GPT-4, and GPT-4V: Can the Large Language Model (ChatGPT) Pass the Japanese Board of Orthopaedic Surgery Examination?

**DOI:** 10.7759/cureus.56402

**Published:** 2024-03-18

**Authors:** Nozomu Nakajima, Takahito Fujimori, Masayuki Furuya, Yuya Kanie, Hirotatsu Imai, Kosuke Kita, Keisuke Uemura, Seiji Okada

**Affiliations:** 1 Orthopaedics, Sakai City Medical Center, Sakai, JPN; 2 Orthopaedic Surgery, Osaka University, Graduate School of Medicine, Suita, JPN

**Keywords:** japanese board of orthopaedic surgery examination, gpt-4v, chatgpt, large language model, artificial intelligence

## Abstract

Introduction

Recently, large-scale language models, such as ChatGPT (OpenAI, San Francisco, CA), have evolved. These models are designed to think and act like humans and possess a broad range of specialized knowledge. GPT-3.5 was reported to be at a level of passing the United States Medical Licensing Examination. Its capabilities continue to evolve, and in October 2023, GPT-4V became available as a model capable of image recognition. Therefore, it is important to know the current performance of these models because they will be soon incorporated into medical practice. We aimed to evaluate the performance of ChatGPT in the field of orthopedic surgery.

Methods

We used three years’ worth of Japanese Board of Orthopaedic Surgery Examinations (JBOSE) conducted in 2021, 2022, and 2023. Questions and their multiple-choice answers were used in their original Japanese form, as was the official examination rubric. We inputted these questions into three versions of ChatGPT: GPT-3.5, GPT-4, and GPT-4V. For image-based questions, we inputted only textual statements for GPT-3.5 and GPT-4, and both image and textual statements for GPT-4V. As the minimum scoring rate acquired to pass is not officially disclosed, it was calculated using publicly available data.

Results

The estimated minimum scoring rate acquired to pass was calculated as 50.1% (43.7-53.8%). For GPT-4, even when answering all questions, including the image-based ones, the percentage of correct answers was 59% (55-61%) and GPT-4 was able to achieve the passing line. When excluding image-based questions, the score reached 67% (63-73%). For GPT-3.5, the percentage was limited to 30% (28-32%), and this version could not pass the examination. There was a significant difference in the performance between GPT-4 and GPT-3.5 (p < 0.001). For image-based questions, the percentage of correct answers was 25% in GPT-3.5, 38% in GPT-4, and 38% in GPT-4V. There was no significant difference in the performance for image-based questions between GPT-4 and GPT-4V.

Conclusions

ChatGPT had enough performance to pass the orthopedic specialist examination. After adding further training data such as images, ChatGPT is expected to be applied to the orthopedics field.

## Introduction

Machines have long been valuable partners to humanity. In our daily lives, we are surrounded by many machines. Modern artificial intelligence (AI), which is attracting significant attention these days, is designed to think and act like humans [1-3]. In the 1970s, attention was drawn to expert systems that aimed to replicate professional advice by teaching machines vast amounts of specialized knowledge [4,5]. A prime example, Mycin, could suggest appropriate antibiotics based on the entered information [6-8]. However, these systems required extensive manual data entry by experts, and due to their rigid rule-based structure, they had limited application [1,9]. Nevertheless, these limitations are being overcome with advancements in machine learning technologies. Introduced by OpenAI (San Francisco, CA) in October 2022, ChatGPT (GPT-3.5) is a prominent large language model. It can instantly generate text based on the vast knowledge acquired from web data and textbooks. It supports multiple languages and is characterized by its conversational skills, making interactions feel like one is speaking to a human [10].

Since GPT-3.5 was released, it has attracted significant attention worldwide for its usefulness, completeness, and high accuracy. As of October 2023, a search for “Large Language Model” in PubMed has yielded 8182 hits, indicating a high level of attention in the medical field. GPT-3.5 was reported to be at a level of passing the United States Medical Licensing Examination (USMLE) [11]. Furthermore, GPT-4, an upgraded model of GPT-3.5, was released in March 2023. Its accuracy was in the top 10% of the US bar exam (compared with the bottom 10% for the GPT-3.5) [10]. This AI is not only capable of natural conversation but also possesses a broad range of specialized knowledge. It is inevitable that, in the future, it will be utilized in the medical field as an AI that can provide expert advice.

In the field of orthopedics, GPT-4 corresponded to the average performance of post-graduate year (PGY)-5 in the Orthopedic In-Training Examination and exceeded the passing score of the American Board of Orthopedic Surgery Part 1 Examination, while GPT-3.5 only corresponded to a PGY-1 level [12]. However, these evaluations were conducted in English, and their performance inputted in Japanese has not yet been assessed. Furthermore, in October 2023, GPT-4V became available, enabling bimodal input through both text and images. No studies have attempted image-based questions using GPT-4V. This study aimed to evaluate the performance of ChatGPT, one of the representative large-scale language models, on the Japanese Board of Orthopaedic Surgery Examination (JBOSE).

## Materials and methods

The Japanese Board of Orthopaedic Surgery Examination (JBOSE)

**Figure 1:**
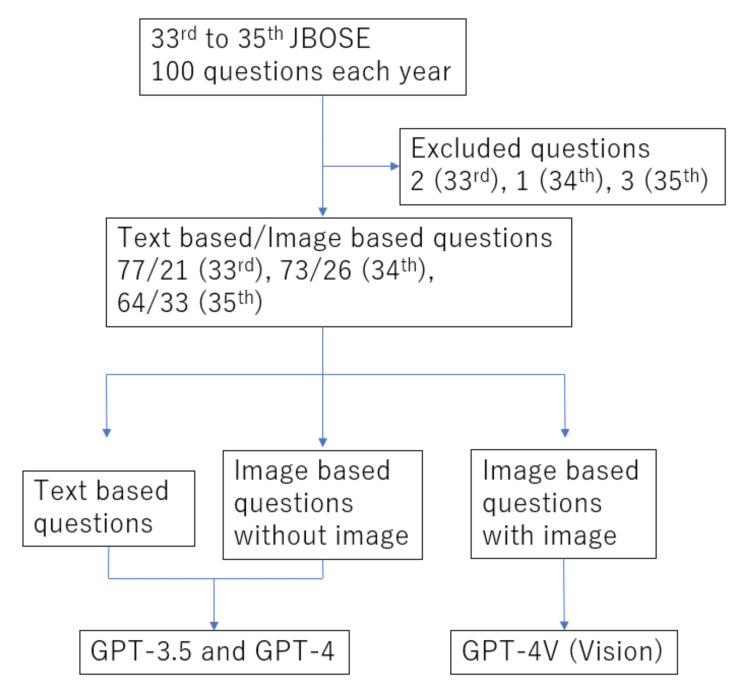
Workflow of this study The Japanese Board of Orthopaedic Surgery Examination (JBOSE) included text-based and image-based questions. A few questions were officially excluded after the examination because of their incompleteness. GPT-4V enables bimodal input through both text and images. The 33rd, 34th, and 35th are ordinal numbers in exams, and they were conducted in 2021, 2022, and 2023, respectively.

This examination is held once a year, consists of 100 questions, and tests a wide range of knowledge related to orthopedics from basic medicine, such as bone metabolism and pathology, to specialized fields, such as conservative therapy, surgical treatment, rehabilitation, and medical insurance. After passing the National Medical Examination in Japan, the applicant must complete two years of initial clinical training, followed by three years and nine months of specialized orthopedic surgery training to be eligible for the examination. The time limit is 120 minutes, and the questions are presented in computer-based testing (CBT) format. Each question consists of a statement and five options. There are three types of questions requiring the selection of one, two, or three correct answers. JBOSE 33rd, 34th, and 35th, conducted in 2021, 2022, and 2023, were used to evaluate the performance of ChatGPT. We focused on the examinations from the past three years because the format changed after the 33rd session, shifting from a combination of written and interview examinations to the CBT format. The questions and correct answers were obtained from the Japanese Orthopaedic Association journal [13-15]. Except for a few questions that were officially announced to be incomplete, all questions were inputted to ChatGPT (Figure 1).

JBOSE includes text-based and image-based questions. Image-based questions include both textual statements and images. Although image information is helpful in predicting the correct answers, it is possible to guess the answer from the textual statements alone, even without images. Because GPT-3.5 and GPT-4 only accept textual input, image-based questions were inputted only as textual statements.

Large language model

The models used in this study were GPT-3.5 (standard model), GPT-4 (high-grade model), and GPT-4V (text and image input model), all of which were large language models developed by OpenAI (San Francisco, CA). While GPT-3.5 was available for free, GPT-4 and GPT-4V required a monthly fee of US$20 as of January 2024. These models generated text based on vast knowledge across various fields and were characterized by their conversational skills, making interactions similar to those of humans. We examined its knowledge in the orthopedic field using JBOSE. The GPT-4V model became available in October 2023. GPT-4V is capable of recognizing images as well as text, therefore we added a verification step to confirm GPT-4V’s performance in recognizing medical images.

Data input

Questions and their multiple-choice answers from the JBOSE were used in their original Japanese form, as was the official examination rubric. Instructions for using ChatGPT were also provided in Japanese. Because GPT-3.5 and GPT-4 were not able to recognize images, we input text data only. In other words, for image-based questions, GPT3.5 and GPT4 answered without images, as a reference, only text data for the questions. We employed prompt engineering techniques to ensure consistency in the interaction and emulation of a clinical examination setting. The following statement was instructed, and then each question was entered one by one: “You are an orthopedic specialist. We will present a test, so please answer the following questions. Make sure to answer each question carefully without any mistakes.” This approach was designed to prime ChatGPT with a specific role and mindset as an orthopedic specialist. When multiple questions were presented at once, there was a risk of information overload and scattered attention to specific questions. Therefore, in each interaction, we inputted questions individually.

Evaluation

To determine whether GPT-3.5 and GPT-4 could pass the orthopedic specialist examination, the accuracy was calculated separately for the entire set of questions and the text-based questions. Accuracy was defined as the percentage of correct answers. As the minimum scoring rate acquired to pass is not officially disclosed, it was determined using publicly available data (total examinees, number of passers, average score, and standard deviation) using the following formula: passing score = Z × standard deviation + average score. Z-score corresponds to the value derived from the standard normal distribution table for “1 − number of passers/total examinees.”

Modified question

To further assess the accuracy of GPT-4, we introduced a “Modified Question” format to the text-based questions. In this approach, the number of correct options was concealed during the question. In other words, the model was instructed to respond without knowing how many correct answers were present among the options. For instance, a question like “Choose the three correct options” would be posed as “Select all the correct options.” The model was assessed on its ability to judge all given options correctly. We presented GPT-4 with modified questions created from the text-based questions of the 33rd to 35th JBOSE and examined its performance and reproducibility. “Reproducibility” was defined as the proportion of questions that were answered correctly in the modified questions out of those that were correctly answered in the conventional questions.

Statistical analysis

Statistical analysis was conducted using Welch's t-test to compare the performance of GPT-3.5 and GPT-4. All statistical tests were two-tailed, and a p-value of less than 0.05 was considered statistically significant. Statistical analysis was performed using SPSS Statistics version 20 software (IBM Corp., Armonk, NY).

## Results

Demographic data

The overview of the examination is shown in Table 1.

**Table 1:** The overview of the 33rd-35th Japanese Board of Orthopaedic Surgery Examination (JBOSE) and the performance of GPT-3.5, GPT-4, and GPT-4V

JBOSE	33rd	34th	35th
Number of examinees (passers/total)	1021/1048	603/653	530/591
Passing rate among all examinees (%)	97	92	90
Number of questions (total/text-based/image-based)	98/77/21	99/73/26	97/64/33
Average score (%)	61	66	64
Standard deviation (%)	9	8.5	8.9
Estimated minimum scoring rate acquired to pass (%)	43.7	53.8	52.7

The estimated minimum scoring rate acquired to pass was calculated as 43.7% for the 33rd, 53.8% for the 34th, and 52.7% for the 35th examination. The pass rate among all examinees is typically around 90% each year. However, the 33rd exam, being the first to adopt the CBT format, saw a higher pass rate of 97%, exceeding the usual average. Subsequently, the pass rates for the 34th and 35th exams were 92% and 90%, respectively. There were 77 (79%) text-based questions and 21 (21%) image-based questions in the 33rd examination, 73 (74%) and 26 (26%) in the 34th examination, and 64 (66%) and 33 (34%) in the 35th examination, respectively.

Performance of each GPT model

For GPT-4, even when answering all questions, including the image-based ones, the accuracy was 60%, 55%, and 61% for each examination. When excluding image-based questions, it was 64%, 63%, and 73%, which was comparable with the average score of examinees. In both cases, the scores achieved the pass line. The accuracy for image-based questions was lower than that for text-based questions. For GPT-4V, the accuracy in image-based questions was 38%, 35%, and 39%, which surpassed that of GPT-4 in the 34th (31%) and 35th (36%) examinations but fell below GPT-4’s performance in the 33rd (48%) examination. For GPT-3.5, the accuracy was limited to 28%, 32%, and 30%, which was unable to achieve a pass in any of the years (Table 2).

**Table 2:** The accuracy of GPT-3.5, GPT-4, and GPT-4V for the 33rd-35th Japanese Board of Orthopaedic Surgery Examination (JBOSE) The accuracy was defined as the percentage of correct answers. All questions include both text-based and image-based questions.

	JBOSE	33rd	34th	35th
		Number of correct answers/total questions, accuracy (%)		
GPT-4	All questions	59/98, (60)	54/99, (55)	59/97, (61)
	Text-based questions	49/77, (64)	46/73, (63)	47/64, (73)
	Image-based questions without image	10/21, (48)	8/26, (31)	12/33, (36)
GPT-4V	Image-based questions with image	8/21, (38)	9/26, (35)	13/33, (39)
GPT-3.5	All questions	27/98, (28)	32/99, (32)	29/97, (30)
	Text-based questions	20/77, (26)	25/73, (34)	23/64, (36)
	Image-based questions without image	7/21, (33)	7/26, (27)	6/33, (18)

For GPT-4, the average accuracies were 59% for all questions, 66% for text-based questions, and 38% for image-based questions. In comparison, GPT-3.5 achieved 30% for all questions, 32% for text-based questions, and 25% for image-based questions. A significant difference was observed between GPT-4 and GPT-3.5 in text-based questions (p = 0.002; Table 3).

**Table 3:** Average of three-year total accuracy of GPT-4, GPT-3.5, and GPT-4V for all questions, text-based questions, and image-based questions The accuracy was defined as the percentage of correct answers. All questions include both text-based and image-based questions. ^*^ p < 0.05. ^† ^p-value was calculated between GPT-4 and GPT-4V. ​​​​​​​^§ ^p-value was calculated between GPT-4 and GPT-3.5. N.A.: not available.

	GPT-4	GPT-3.5	GPT-4V	p-value
	Number of correct answers/total questions, accuracy (%)			
All questions	172/294, (59)	88/294, (30)	N.A.	<0.001^*§^
Text-based questions	142/214, (66)	68/214, (32)	N.A.	0.002^*§^
Image-based questions without image	30/80, (38)	20/80, (25)	N.A.	0.1^§^
Image-based questions with image	N.A.	N.A.	30/80, (38)	0.9^†^

Performance of GPT-4 for modified question

**Figure 2:**
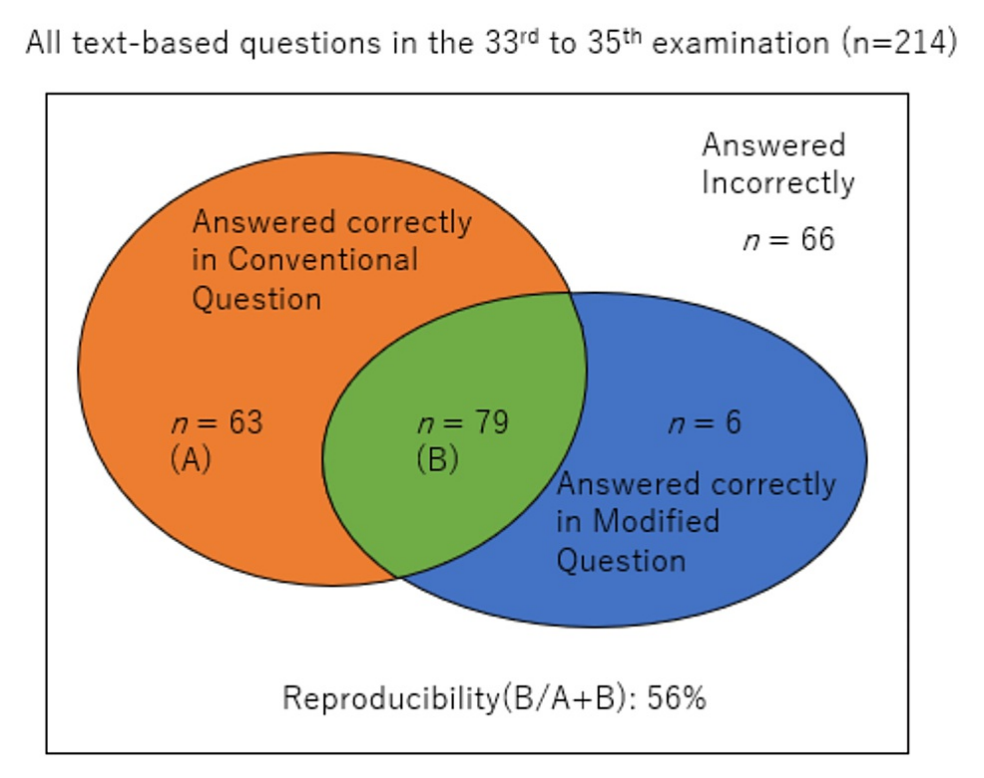
GPT-4’s performance on the modified question and its reproducibility To further assess the performance of GPT-4, a “Modified Question” was made for all text-based questions in the 33rd-35th examination (n = 214), concealing the number of correct options. Reproducibility was defined as the proportion of questions answered correctly in the modified question format (A) out of those correctly answered in the conventional question format (A+B).

We made ChatGPT answer the modified questions that had no information about the number of correct options. Modified questions were made for all text-based questions in the 33rd to 35th examination (n = 214). Out of the questions correctly answered in the conventional questions (n = 142), 79 were also correctly answered in the modified questions, resulting in a reproducibility of 56%. Additionally, there were a few questions (n = 6) answered correctly in the modified questions, but answered incorrectly in the conventional questions (Figure 2).

## Discussion

**Figure 3:**
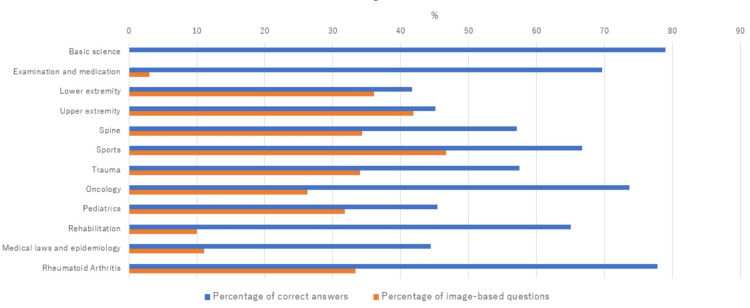
Percentage of correct answers and image-based questions categorized by subject type The percentage of correct answers on GPT-4 for all questions was categorized by subject type. There was a tendency for the accuracy rate to increase with a lower proportion of image-based questions.

We evaluated ChatGPT's performance in the orthopedic field using the JBOSE. While GPT-3.5 did not reach the passing line for the exam, GPT-4 achieved the passing line despite including image-based questions. Although GPT-4 could not recognize the image, the GPT-4’s accuracy for the image-based questions reached 38%. This fact suggests that GPT-4 may have advanced reasoning skills. It has been reported that GPT-4’s advancements were not merely due to increased training data; it can now interpret complex texts and grasp nuanced differences [10]. We think that GPT-4 was able to deduce correct answers even from incomplete questions using its clinical reasoning skills. The percentage of correct answers on GPT-4 for all questions was categorized by subject type (Figure 3).

While the accuracy rate was higher for the basic science, which asked for simple knowledge, it was lower for questions involving image-based questions.

**Figure 4:**
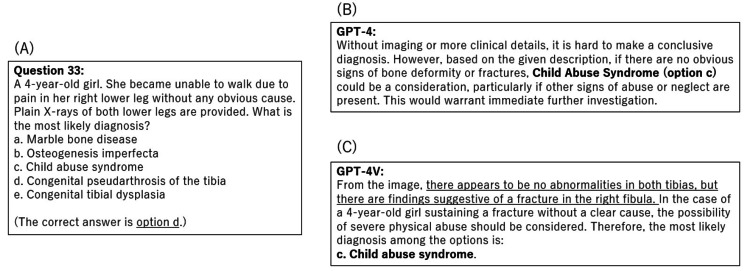
The examples of responses of GPT-4 and GPT-4V to image-based questions. Even if images were provided to GPT-4V, the responses were similar to GPT-4, in which no image was provided. (A) An example of a question that includes both textual statements and images. X-ray images were omitted for copyright reasons. The textual statement was translated from Japanese. (B) The answer obtained from GPT-4. (C) The answer obtained from GPT-4V. Image credits: Nozomu Nakajima.

Since October 2023, GPT-4V has become available and features image recognition capabilities. However, the accuracy of image-based questions showed no improvement. There was a tendency for questions incorrectly answered by GPT-4 to also be answered incorrectly by GPT-4V. Specific examples of responses by GPT-4 and GPT-4V to image-based questions are shown in Figure 4.

While the presence of a fracture was recognized with the inclusion of an image, GPT-4V failed to identify the specific location of the fracture or the characteristic changes necessary for diagnosis. Consequently, the responses were similar to those given when no image was provided. We think that GPT-4V has been trained sufficiently for medical imaging due to the difficulties in acquiring extensive image data, given the concerns over personal privacy.

The utility of ChatGPT in the medical field is gathering attention, and several existing studies support this notion [11,12,16-21]. We summarized in Table 4 research compiling examinations that evaluated knowledge using ChatGPT.

**Table 4:** The summary of existing studies compiling examinations that evaluate knowledge using ChatGPT

	Author (year)	Exam	Language	Model	Result
General Medicine	Kung et al. (2023) [11]	United States Medical Licensing Examination	English	GPT-3.5	Pass
	Takagi et al. (2023) [16]	Japanese Medical Licensing Examination	Japanese	GPT-4	Pass
Orthopedics	Kung et al. (2023) [12]	Orthopaedic In-Training Examination	English	GPT-4	Pass
				GPT-3.5	Fail
	Saad et al. (2023) [17]	Orthopaedic Fellow of the Royal College of Surgeons	English	GPT-4	Fail
	Massey et al. (2023) [18]	ResStudy Orthopaedic Examination Question Bank	English	GPT-4	Fail
Neurosurgery	Ali et al. (2023) [19]	Neurosurgery Written Board Examinations	English	GPT-4	Pass
				GPT-3.5	Pass
Gastroenterology	Suchman et al. (2023) [20]	American College of Gastroenterology Self-Assessment Test	English	GPT-4	Fail
Radiology	Bhayana et al. (2023) [21]	Canadian Royal College and American Board of Radiology examinations	English	GPT-3.5	Pass

While there were slight variations in the prompts, we found that GPT-3.5 could pass the USMLE [11]. However, one of the notable limitations of GPT-3.5 was its lower accuracy in non-English languages [22], which was particularly evident in the Japanese medical licensing exam where GPT-3.5 could not achieve a pass level. In contrast, GPT-4 showed significant improvement, successfully passing the same exam [16]. This enhancement in GPT-4’s performance in non-English languages marks a critical step in its applicability in global medical contexts. In studies of specialties, most of the exams were administered in English. It was reported that ChatGPT achieved the passing line in orthopedics, neurosurgery, and radiology examinations [12,19,21]. On the other hand, some reports reported that ChatGPT has not yet reached the level of a specialist [17,18,20]. Our study indicated that GPT-4 may have knowledge at the level of the Japanese license of an orthopedic specialist who has trained for five years and nine months after obtaining their medical license [10,23]. Although it is not possible to make a simple comparison with examinations in English, as the difficulty level and the passing line are different, it is apparent that ChatGPT has reached a certain level in the Japanese orthopedic field.

**Figure 5:**
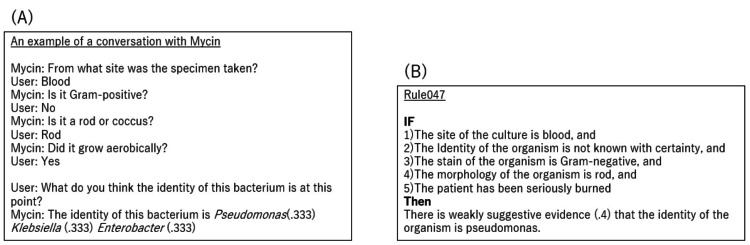
An example of a conversation with Mycin To enter the characteristics of the patient, Mycin can guess the identity of bacteria and suggest appropriate antibiotics from the 200 rules. (A) An example of a conversation with Mycin. (B) One example out of 200 rules. Image credits: Nozomu Nakajima.

Expert systems, developed in the 1970s, stood as AI designed to mimic human knowledge in specific domains. These systems aimed to emulate experts by absorbing vast amounts of precise information. Mycin was the typical model. It could guess the identity of bacteria and suggest appropriate antibiotics from the programmed 200 rules by entering the characteristics of the patient (Figure 5) [8].

However, despite the innovative system, they were not widely used because they required experts to manually input knowledge, making updates difficult. Additionally, being rule-based, these systems could only process inputs in a predetermined manner, restricting their adaptability [4,5]. In contrast, ChatGPT boasts a wide-ranging knowledge base, allowing for flexible, rapid responses in conversational formats, highlighting its superiority [23,24]. While remaining adaptable, ChatGPT has rapidly improved in accuracy to a level that would pass the JBOSE [25]. ChatGPT has the potential to evolve into a state-of-the-art expert system by further increasing knowledge in specialized areas.

In addition to issues like response reliability, a challenge known as “Artificial Hallucination” is highlighted. This refers to AI generating incorrect or unsubstantiated information as if it were factual, a significant concern with large language models [26,27]. While the rate of hallucinations in GPT-4 is reportedly decreasing, it is still unsatisfactory [28]. These hallucinations, essentially AI “misconceptions” based on false information, can be hard to discern without expert knowledge. This leads to potential misreading and underscores the need for further refinement.

Although GPT-4 corresponded to the passing level, the performance on modified questions (where the number of correct options was concealed) was lower compared with conventional questions. In some cases, once a question was answered correctly, it would respond incorrectly when asked at a different time. There was some randomness in ChatGPT’s responses because it is based on transformer architecture, which works by predicting the next word in a sequence based on probabilities. It analyzes the context and previous words to estimate which word might logically follow. This process, drawing from extensive training data, results in some unpredictability or randomness in the responses [10,24].

This study had some limitations. First, ChatGPT is continuously evolving through user feedback, which means that outcomes might vary depending on the timing of the test. The evaluations of GPT-3.5 and GPT-4 were conducted in August 2023, while GPT-4V was tested in October. Similarly, the randomness of ChatGPT's responses could also affect the results, but each model was tested only once in this test. Second, the quality of images of image-based questions was not very high. For this study, images were downloaded as monochrome PDFs from the society's website. However, in the actual examination, more high-quality color images are available on computer screens. This may lead to GPT-4V’s failure to produce a better performance than GPT-4 in the image-based questions.

## Conclusions

This study demonstrated that ChatGPT has the performance to pass the Japanese orthopedic specialist examination. However, even if ChatGPT can pass the exam, it cannot replace the clinician as is. "Artificial hallucinations" can cause harm to patients, and the use of such technology in medical practice should be carefully phased in.

However, with its extensive knowledge across a wide range of fields beyond medicine, the user-friendly chat format, and multimodal functionalities like image recognition and web search, ChatGPT has the potential to be a potent support tool. Therefore, we continuously need to evaluate and improve the evolving capabilities of AI to ensure its beneficial and safe use in the medical field.
